# Co-delivery of autophagy inhibitor and gemcitabine using a pH-activatable core-shell nanobomb inhibits pancreatic cancer progression and metastasis

**DOI:** 10.7150/thno.60437

**Published:** 2021-08-04

**Authors:** Xiaoxiao Chen, Yuan Tao, Manmin He, Miao Deng, Rong Guo, Qinglin Sheng, Xuhui Wang, Kebai Ren, Ting Li, Xuan He, Shuya Zang, Zhirong Zhang, Man Li, Qin He

**Affiliations:** Key Laboratory of Drug-Targeting and Drug Delivery System of the Education Ministry and Sichuan Province, Sichuan Engineering Laboratory for Plant-Sourced Drug and Sichuan Research Center for Drug Precision Industrial Technology, West China School of Pharmacy, Sichuan University, Chengdu 610041, People's Republic of China.

**Keywords:** autophagy inhibition, pancreatic cancer, size-shrinkage, tumor metastasis, deep penetration

## Abstract

**Background:** Metastasis is one of the main reasons for the high mortality associated with pancreatic ductal adenocarcinoma (PDAC), and autophagy regulates the metastatic migration of tumor cells, their invasion of tissues, and their formation of focal adhesions. Inhibiting autophagy may suppress tumor growth and metastasis, but the abundant extracellular matrix hinders the deep penetration of therapeutic agents.

**Methods:** To enhance the penetration of drugs that can inhibit metastasis of pancreatic cancer, a pH-responsive drug delivery system was formulated. Gemcitabine (GEM), a first-line chemotherapeutic drug against PDAC, was loaded in 6PA-modified DGL (PDGL) nanoparticles to afford PDGL-GEM. Then PDGL-GEM was co-precipitated with the autophagy inhibitor chloroquine phosphate (CQ) and calcium phosphate to formulate PDGL-GEM@CAP/CQ. The size and morphology of the resulting “nanobomb” PDGL-GEM@CAP/CQ were characterized, and their uptake into cells, cytotoxicity and ability to inhibit autophagy were analyzed at pH 6.5 and 7.4. The anti-tumor and anti-metastasis effects of the nanobomb were explored on mice carrying Pan 02 pancreatic tumor xenografts or orthotopic tumors.

**Results:** The pH-induced dissolution of calcium phosphate facilitated the release of CQ from the nanobomb and deep penetration of PDGL-GEM. The internalization of PDGL-GEM and subsequent intracellular release of GEM inhibited tumor growth, while CQ downregulated autophagy in tumor cells and fibroblasts. In fact, inhibition of xenograft and orthotopic tumor growth was greater with the complete PDGL-GEM@CAP/CQ than with subassemblies lacking GEM or CQ. More importantly, mechanistic studies *in vitro* and *in vivo* suggested that the nanobomb inhibits metastasis by downregulating MMP-2 and paxillin, as well as reducing fibrosis.

**Conclusion:** The pH-sensitive PDGL-GEM@CAP/CQ shows potential for inhibiting proliferation and metastasis of pancreatic cancer through an autophagy-dependent pathway.

## Introduction

Pancreatic ductal adenocarcinoma (PDAC), generally characterized by proliferation of desmoplastic stromal cells and metabolic dysfunction, is considered one of the most aggressive cancers and is associated with high rates of metastasis and mortality 1, 2. The 5-year survival rate of PDAC remains below 10% 3-5. The symptoms of early-stage disease are non-specific, making timely diagnosis difficult. By the time it has been diagnosed, PDAC has usually metastasized to multiple organs, most often the liver and peritoneum, making surgical treatment impossible. Effective strategies against PDAC onset and metastasis are urgently needed.

A useful strategy may be to target autophagy, a homeostatic mechanism through which cells degrade senescent organelles, pathogens and proteins. Autophagy plays essential roles in PDAC initiation and progression 7-9, and it is up-regulated in tumor cells and cells in the tumor stroma 6. Up-regulated autophagy facilitates tumor metastasis by degrading paxillin, which disassembles focal adhesions and promotes tumor mobility 10-12. Autophagy also promotes immune evasion by PDAC tumors 6, 13. The activated pancreatic stellate cells of patients with pancreatic cancer express high levels of autophagy markers, extracellular matrix (ECM) proteins and interleukin-6 (IL-6), and the greater production of these proteins is associated with worse prognosis, shorter survival and higher risk of recurrence 14, 15.

The combination of autophagy inhibitors and chemotherapy or inhibitors of extracellular signal-regulated kinase pathway has been reported to inhibit PDAC tumors in an animal model 16, 17. Autophagy inhibitors can induce pancreatic stellate cells to enter a quiescent state that secretes less IL-6, consequently inhibiting tumor metastasis 15. Therefore, inhibiting the level of autophagy in the tumor microenvironment (TME) shows promise for strengthening the anti-tumor and anti-metastatic effects of pancreatic cancer chemotherapy.

However, treating PDAC is challenging because the dense stroma makes it difficult to deliver drugs to the TME and the tumor itself. The stroma comprises cancer-associated fibroblasts, which contain pancreatic stellate cells, stromal cells, mesenchymal stem cells and immune cells, together with secreted ECM proteins. The stroma makes up to 90% of the total mass of pancreatic cancer, and it forms a physical and metabolic barrier against therapeutics 18, 19. To circumvent these barriers, size-changeable drug delivery systems have been developed that can target different cell types within the TME 20-22. Generally, nanoparticles larger than 100 nm are not cleared rapidly but instead are retained in the TME, while nanoparticles of 10-30 nm penetrate deep into the tumor, which is important for PDAC treatment 23. In previous work, we conjugated dendrigraft poly-L-lysine (DGL) with poly(ethylene glycol)-poly(caprolactone) (PEG-PCL) micelles to create a matrix metalloproteinase (MMP)-responsive nanoparticle that released drug-loaded DGL for deep penetration into the TME 24, 25. Encouraged by these findings, we hypothesized that a TME-responsive, size-shrinkable, sequential delivery system could penetrate the TME to suppress PDAC growth and metastasis.

We planned to constitute such a delivery system using calcium phosphate (CAP), a natural biological material that is the basic component of bones and teeth, and that therefore has good biocompatibility and biodegradability 26. We chose chloroquine phosphate (CQ) as autophagy inhibitor because phosphate-containing drugs have a similar structure as CAP and therefore the two species can co-precipitate during formulation 12, 27. The pH sensitivity allows CAP to degrade in the acidic TME, thereby releasing CQ to inhibit autophagy in tumor cells and stromal cells.

We formulated our delivery system by modifying DGL with 6-phosphonohexanoic acid (6PA) to generate PDGL, which we loaded with the first-line PDAC drug gemcitabine (GEM) to yield PDGL-GEM. Then we co-precipitated the PDGL-GEM, CQ and CAP, which we stabilized using PEG_5k_. In the acidic TME, CAP disintegrates in a “bomb-like” process to release CQ and PDGL-GEM. CQ can be internalized by surrounding fibroblasts, where it inhibits autophagy to reduce fibrosis. Simultaneously, the small DGL-GEM can penetrate deep into the tumor. The present study is the first report of a TME-sensitive dual targeting strategy for reprogramming the TME to enhance PDAC chemotherapy, providing a new perspective for treatment.

## Results and Discussion

### Preparation and characterization of PDGL-GEM@CAP/CQ

**Figure 1:**
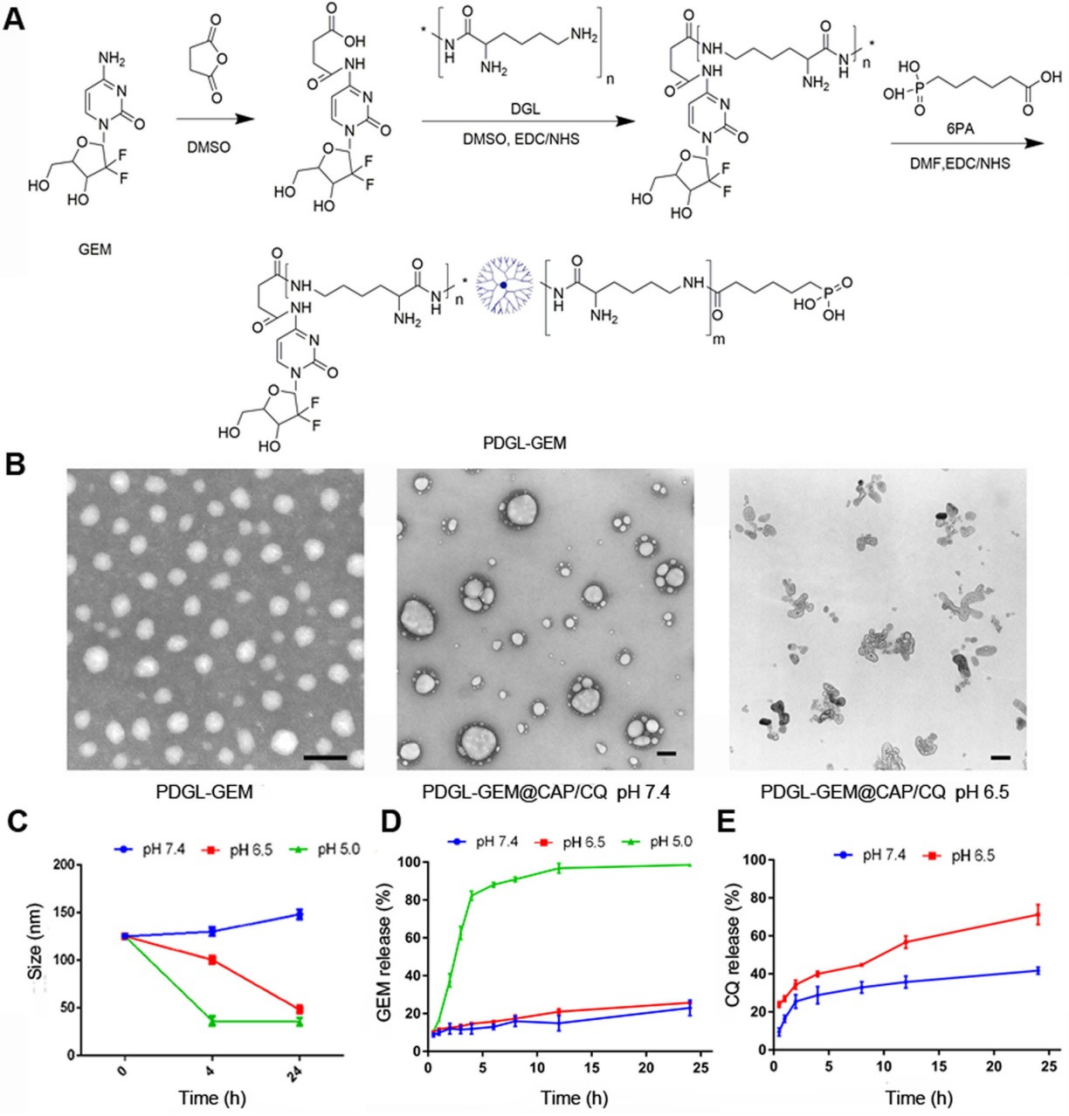
** (A)** Schematic of PDGL-GEM synthesis. **(B)** Transmission electron micrographs of PDGL-GEM and of PDGL-GEM@CAP/CQ at pH 6.5 or 7.4. Scale bar: 100 nm. **(C)** Size of PDGL-GEM@CAP/CQ incubated at different pHs (n = 3, mean ± SD). **(D)** Release of GEM and CQ from PDGL-GEM@CAP/CQ at different pHs (n = 3, mean ± SD).

DGL-GEM was synthesized based on our previous work 25, and the synthesis of DGL-GEM was confirmed by ^1^H-NMR (Figure S1). We conjugated 6PA to the remaining amino groups of DGL-GEM via an amide reaction, generating PDGL-GEM (Figure 1A). Synthesis of PDGL-GEM was confirmed using ^1^H-NMR (Figure S2). PDGL-GEM was dissolved in 1 mL HEPES-NaCl buffer together with CQ and the stabilizer phosphate-PEG_5k._ An equal volume of 1 mM Tris-HCl (pH 7.4) containing 300 mM CaCl_2_ was added dropwise under stirring at 1000 rpm at 40 °C to formulate the PDGL-GEM@CAP/CQ co-precipitate.

Dynamic light scattering indicated that the PDGL-GEM dendritic nanoparticles had a size of 33.5 ± 1.15 nm and a polydispersity index of 0.287 ± 0.024. Precipitation with CAP alone increased particle size to approximately 110 nm, while co-precipitation with CAP and CQ further increased the size to 125.25 ± 1.06 nm (Table 1). Transmission electron microscopy revealed PDGL-GEM to be spherical, with a size consistent with that determined by dynamic light scattering. In contrast, the final PDGL-GEM@CAP/CQ nanoparticles showed a near-spherical structure, and incubating them at pH 6.5 caused the nanoparticles to disassemble (Figure 1B). The loading capacities of the final nanoparticles were 6.42 ± 0.58% for GEM and 30.52 ± 3.56% for CQ.

We confirmed that acidic pH induced the release of GEM and CQ from PDGL-GEM@CAP/CQ nanoparticles. Whereas the nanoparticles remained stable for at least 24 h at pH 7.4 (mimicking the physiological environment), their size decreased over the same period to about 30 nm at pH 6.5 (mimicking the acidic TME), due to the gradual dissolution of CAP. The same extent of shrinkage was achieved in only 4 h by incubating the nanoparticles at pH 5.0 (mimicking the lysosomal environment), reflecting complete dissolution of the CAP shell (Figure 1C). This dissolution was associated with release of GEM, based on high-performance liquid chromatography (Figure 1D). GEM was released slowly at pH 7.4 and 6.5, but rapidly at pH 5.0. In contrast, more than 60% of CQ was released quickly from the CAP shell at pH 6.5. These results suggest that PDGL-GEM@CAP/CQ is stable in a physiological environment and sustainably releases GEM and CQ in response to acidic pH.

PDGL-GEM@CAP/CQ remained well dispersed, without aggregation, after incubation for up to 24 h in 50% fetal bovine serum (Figure S3A). Encapsulating PDGL within CAP substantially reduced the ability of PDGL to lyse red blood cells, suggesting that PDGL-GEM@CAP/CQ has good biocompatibility (Figure S3B-C).

### Uptake of PDGL@CAP by PDAC cells in culture

**Figure 2:**
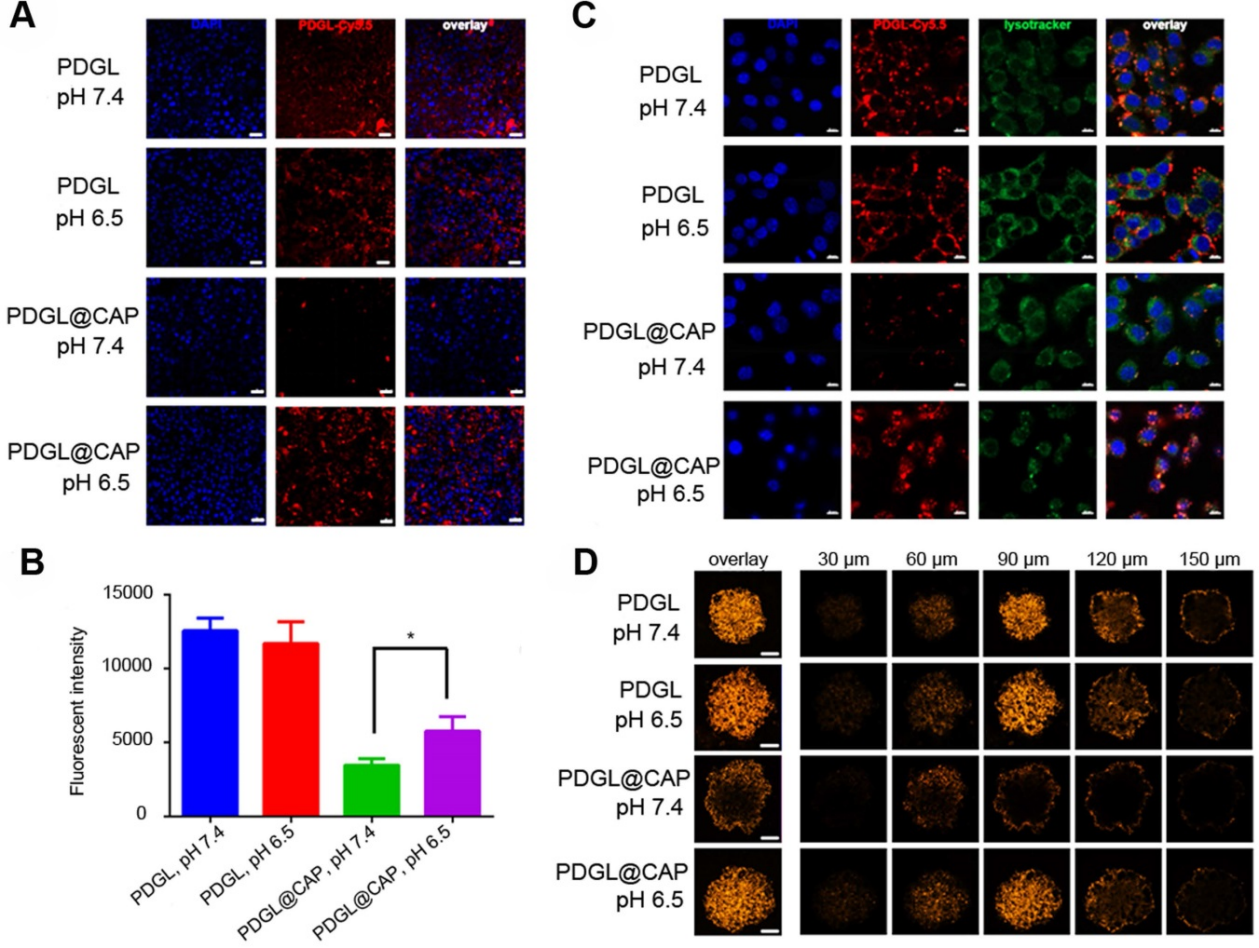
** (A)** Confocal micrographs showing uptake of Cy5.5-labeled nanoparticles by Pan 02 cells. Scale bar: 20 µm. **(B)** Uptake of Cy5.5-labeled nanoparticles by Pan 02 cells, as measured by flow cytometry (n = 3, mean ± SD). * *p* < 0.05. **(C)** Colocalization of Cy5.5-labeled nanoparticles (red) and lysosomes (green). Nuclei were stained with DAPI (blue). Scale bar: 10 µm. (D) Penetration of Cy5.5-labeled nanoparticles into 3D Pan 02 tumor spheroids. Scale bar: 100 µm.

Uptake of Cy5.5-labeled nanoparticles by the PDAC cell line Pan 02 was measured by flow cytometry and confocal laser scanning microscopy. Regardless of pH, small PDGL was efficiently internalized (Figure 2A-B). PDGL@CAP was internalized nearly twice as much at pH 6.5 as at pH 7.4. We attribute this to CAP dissolution, which releases small PDGL and is more easily internalized. Mechanistic experiments suggested that Cy5.5-PDGL@CAP was internalized mainly by a caveola-associated pathway (Figure S4). At pH 6.5, Cy5.5-labelled PDGL@CAP was degraded to release Cy5.5-PDGL, which was internalized through caveola-mediated endocytosis as well as micropinocytosis.

We studied the colocalization of Cy5.5-labeled nanoparticles (red) and lysosomes (green) using confocal laser scanning microscopy (Figure 2C). Compared to the results at pH 7.4, PDGL@CAP was taken up to a greater extent at pH 6.5, and the nanoparticles were more dispersed in the cytoplasm. In contrast, PDGL alone tended to accumulate in lysososomes at both pHs. Our results suggest that the CAP structure facilitates endosomal escape. We hypothesize that the high H^+^ concentration in endolysosomes interrupts the coordination between calcium and phosphate ions in CAP, leading to nanoparticle dissociation and endosomal escape 28.

To explore the ability of PDGL@CAP to penetrate deeply into tumors, we analyzed nanoparticle uptake into Pan 02 tumor spheroids (Figure 2D). PDGL penetrated to a depth of 150 nm at both pH 6.5 and 7.4, probably owing to its small size. The larger PDGL@CAP penetrated to a small extent at pH 7.4, whereas it penetrated to a similar extent as the much smaller PDGL at pH 6.5. These results support our idea that the acidity of the TME induces nanoparticle shrinkage, which in turn facilitates deep tumor penetration.

### Toxicity of PDGL-GEM@CAP/CQ against PDAC cells in culture

**Figure 3:**
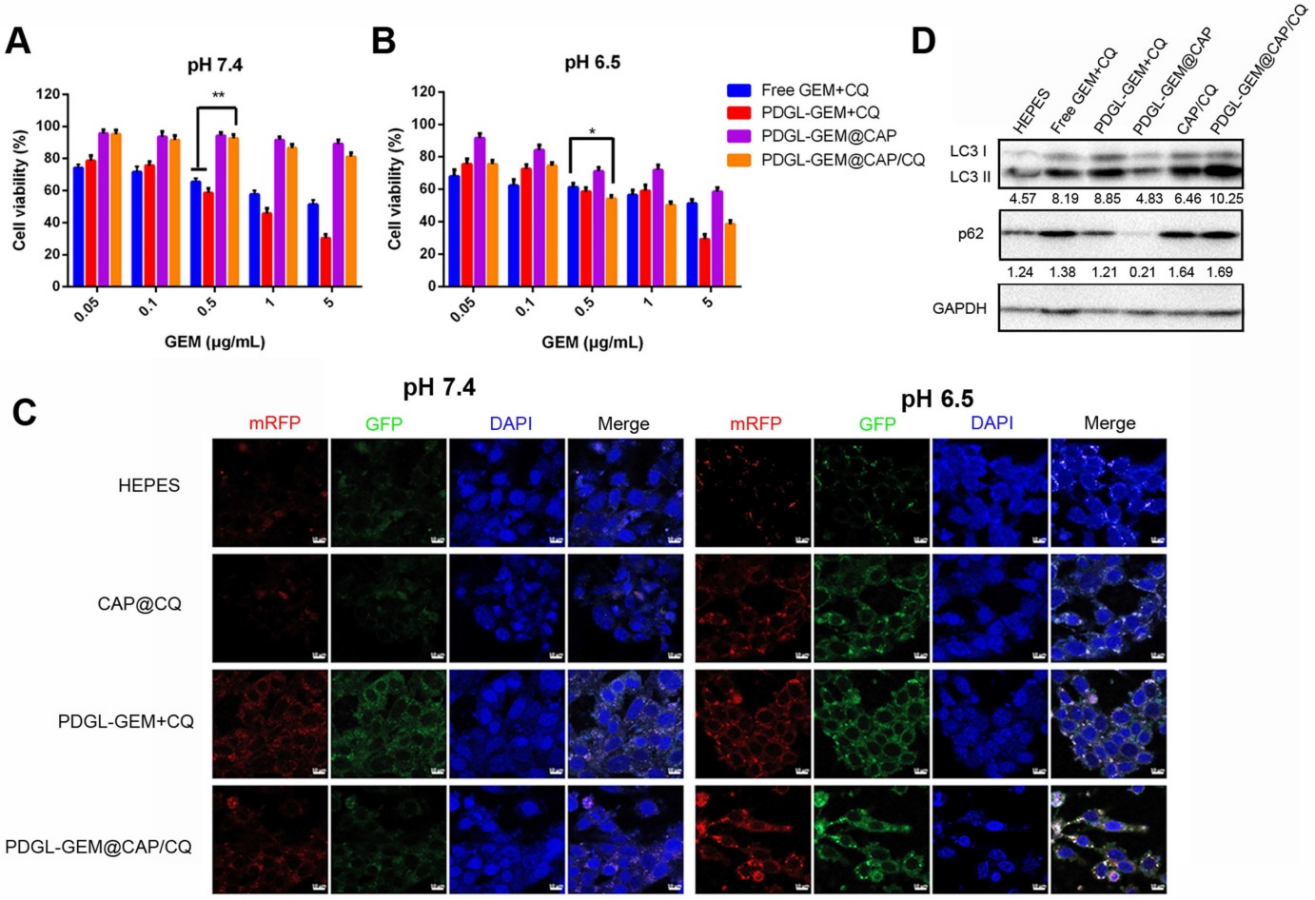
Toxicity of different nanoparticle formulations against Pan 02 cells at **(A)** pH 7.4 or **(B)** pH 6.5 (n = 5, mean ± SD). * *p* < 0.05, ** *p* < 0.01. **(C)** Representative confocal micrographs of Pan 02 cells expressing mRFP-GFP-LC3 and treated for 4 h with the indicated nanoparticles at different pHs. Scale bar: 10 µm. **(D)** Levels of LC3 I, LC 3 II and p62 in Pan 02 cells incubated with the indicated nanoparticles at pH 6.5, based on western blot. Relative expression levels beneath the gel images were determined using Image J. Levels of p62 were normalized to those of GAPDH, and levels of LC3 II were normalized to those of LC3 I.

PDGL was more toxic to fibroblast cells NIH3T3 than CAP, PDGL-CAP or PEGylated PDGL-CAP (PDGL@CAP), probably due to the high positive charge (Figure S5). PEGylation of PDGL@CAP reduced its cytotoxicity substantially, leading to > 80% viability at concentrations as high as 100 μg/mL. Next we investigated the toxicity of a simple mixture of GEM and CQ or drug-loaded nanoparticles against Pan 02 cells (Figure 3A-B). At pH 6.5 and 7.4, PDGL-GEM@CAP/CQ was less toxic than either the simple mixture of GEM and CQ, or small PDGL-GEM + CQ, probably due to greater internalization of the latter two. The CAP-containing nanoparticles, PDGL-GEM@CAP and PDGL-GEM@CAP/CQ, showed no apparent cytotoxicity at pH 7.4 but substantial toxicity at pH 6.5, owing to CAP degradation in the acidic environment and PDGL-GEM release. At pH 6.5, PDGL-GEM@CAP/CQ was similarly cytotoxic as PDGL-GEM + CQ, indicating that PDGL-GEM retains its cytotoxicity after being released from PDGL-GEM@CAP/CQ under acidic conditions. PDGL-GEM@CAP/CQ proved more cytotoxic than PDGL-GEM@CAP, indicating synergistic anti-tumor effects of GEM and CQ.

### Autophagy inhibition by PDGL-GEM@CAP/CQ in PDAC cells in culture

To observe the effects of nanoparticles on autophagy, we labeled autophagosomes by transfecting Pan 02 cells with a plasmid expressing monomeric red fluorescent protein (mRFP), green fluorescent protein (GFP) and the autophagy protein LC3. When autophagosomes fuse with lysosomes, the acid-sensitive GFP is quenched, and the vesicles appear red. In contrast, when autophagy is inhibited, such as in the presence of CQ, then autophagosomes accumulate, leading to a yellow overlay of red and green fluorescence. Cells treated with PDGL-GEM + CQ showed yellow fluorescence at pH 6.5 and 7.4 (Figure 3C), confirming the ability of CQ to inhibit autophagy, presumably by rendering lysosomes alkaline. Treating cells with CAP/CQ or PDGL-GEM@CAP/CQ led to strong yellow fluorescence at pH 6.5 but not at pH 7.4. We hypothesize that acid-induced CAP degradation enhanced CQ internalization, leading to accumulation of autophagosomes. PDGL-GEM@CAP/CQ led to stronger yellow fluorescence than CAP/CQ at pH 6.5, indicating that the dissolution of CAP promotes CQ release autophagy inhibition. This probably reflects that the relatively large PDGL-GEM@CAP/CQ at pH 7.4 was not efficiently internalized, whereas the acid-induced dissociation into PDGL-GEM and CQ led to efficient internalization at pH 6.5.

Western blotting of autophagy-related proteins confirmed the efficient inhibition of autophagy by PDGL-GEM@CAP/CQ. Treating cells with these nanoparticles at pH 6.5 led to the highest ratio of LC3 II to LC3 I and the highest levels of p62, indicating the greatest accumulation of autophagosomes (Figure 3D).

Consistent with these live-cell microscopy and western blotting experiments, we found by transmission electron microscopy that treating cells with PDGL-GEM@CAP/CQ led to strong accumulation of autophagosomes at pH 6.5, but not at pH 7.4 (Figure S6). Together, all these *in vitro* experiments suggest that PDGL-GEM@CAP/CQ inhibits autophagy in PDAC cells in response to the acidity of the TME.

### Inhibition of PDAC cell migration by PDGL-GEM@CAP/CQ in culture

**Figure 4:**
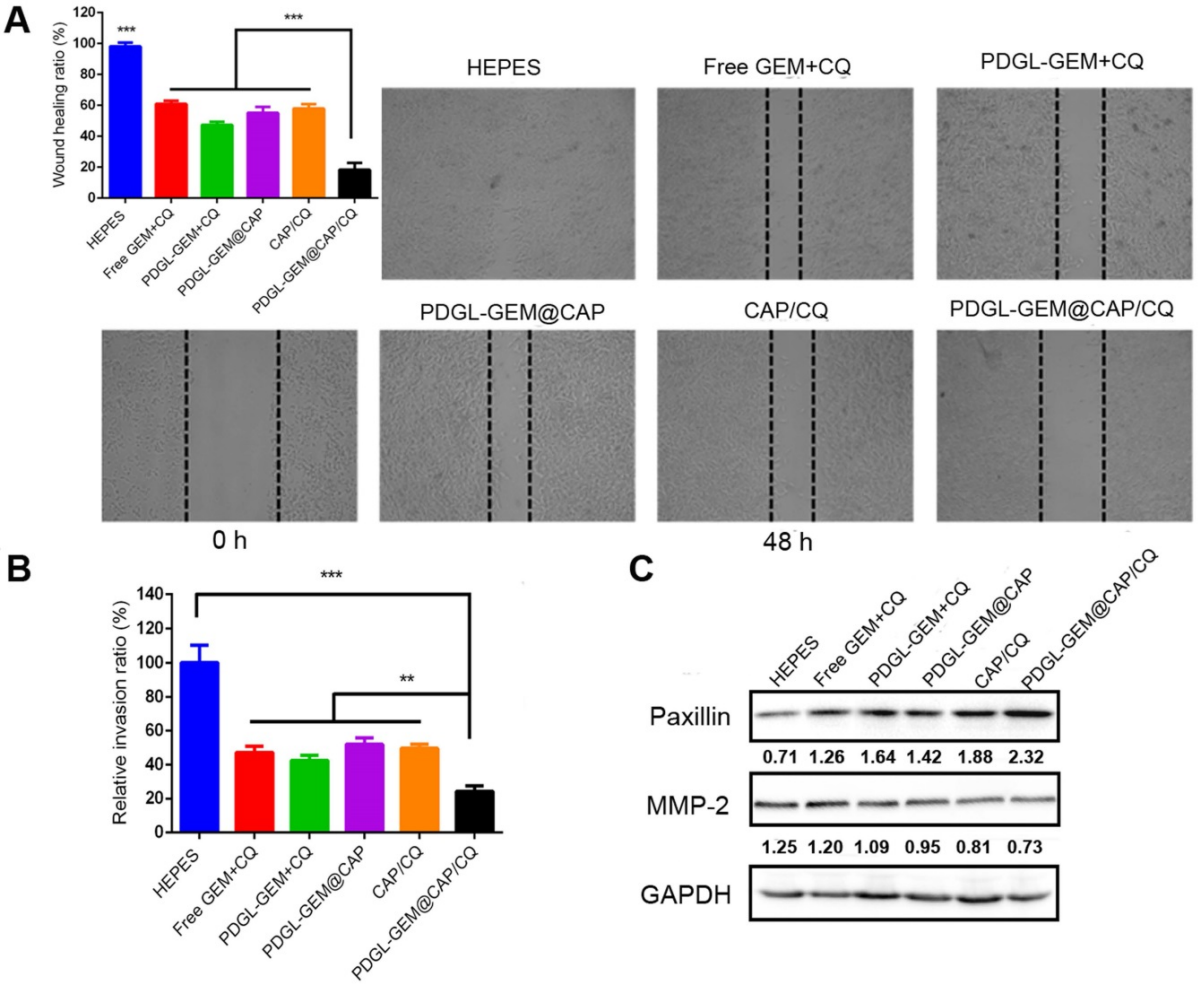
Ability of different nanoparticles to inhibit migration or invasion of Pan 02 cells in **(A)** a wound healing assay or **(B)** a Matrigel-coated invasion assay. Results were normalized to those obtained for the untreated culture or HEPES control. Cells were incubated with the indicated preparations for 48 h. Data are mean ± SD (n = 3). ** *p* < 0.01, *** p < 0.001. Representative images are shown for each group. **(C)** Levels of paxillin and MMP-2 in Pan 02 cells at pH 6.5, as detected by western blot. Relative expression levels beneath the gel images were determined using Image J. Levels of p62 were normalized to those of GAPDH, and levels of LC3 II were normalized to those of LC3 I. The semi-quantitative results represented the gray values measured by image J and normalized to GAPDH.

The ability of PDGL-GEM@CAP/CQ to inhibit the migration of pancreatic cancer cells was tested *in vitro* using a wound healing assay (Figure 4A). PDGL-GEM + CQ inhibited wound healing more than a simple mixture of GEM and CQ, presumably by increasing internalization of the drug. PDGL-GEM@CAP/CQ led to < 20% of the healing observed in untreated cells, presumably reflecting the synergistic effect of the autophagy inhibitor CQ and chemotherapeutic agent PDGL-GEM. Indeed, PDGL-GEM@CAP/CQ led to significantly less healing than PDGL-GEM@CAP or CAP/CQ.

As an additional *in vitro* test of the ability of PDGL-GEM@CAP to inhibit PDAC migration, we simulated the peritumor stroma using a Matrigel-coated Transwell chamber. At pH 6.5, PDGL-GEM@CAP/CQ inhibited migration to a greater extent than CAP/CQ or PDGL-GEM@CAP (Figures 4B and S7), reflecting the dual action of CQ and GEM.

To begin to understand how PDGL-GEM@CAP/CQ may inhibit PDAC metastasis, we explored levels of paxillin and MMP-2 in cells treated with different nanoparticles. Paxillin is a critical component of the adhesion plaques that hold cells within the primary tumor; degradation of paxillin is associated with increased metastasis 11, 29. MMP-2 degrades ECM, so its up-regulation has been linked to metastatic behavior. PDGL-GEM@CAP/CQ preserved paxillin levels and down-regulated MMP-2 more than CAP/CQ, PDGL-GEM@CAP or PDGL-GEM + CQ did (Figure 4C). These results suggest that PDGL-GEM@CAP/CQ inhibits tumor cell migration and invasion by inhibiting the degradation of paxillin and down-regulating MMP-2.

### Ability of PDGL@CAP to accumulate in PDAC tumors *in vivo*

**Figure 5:**
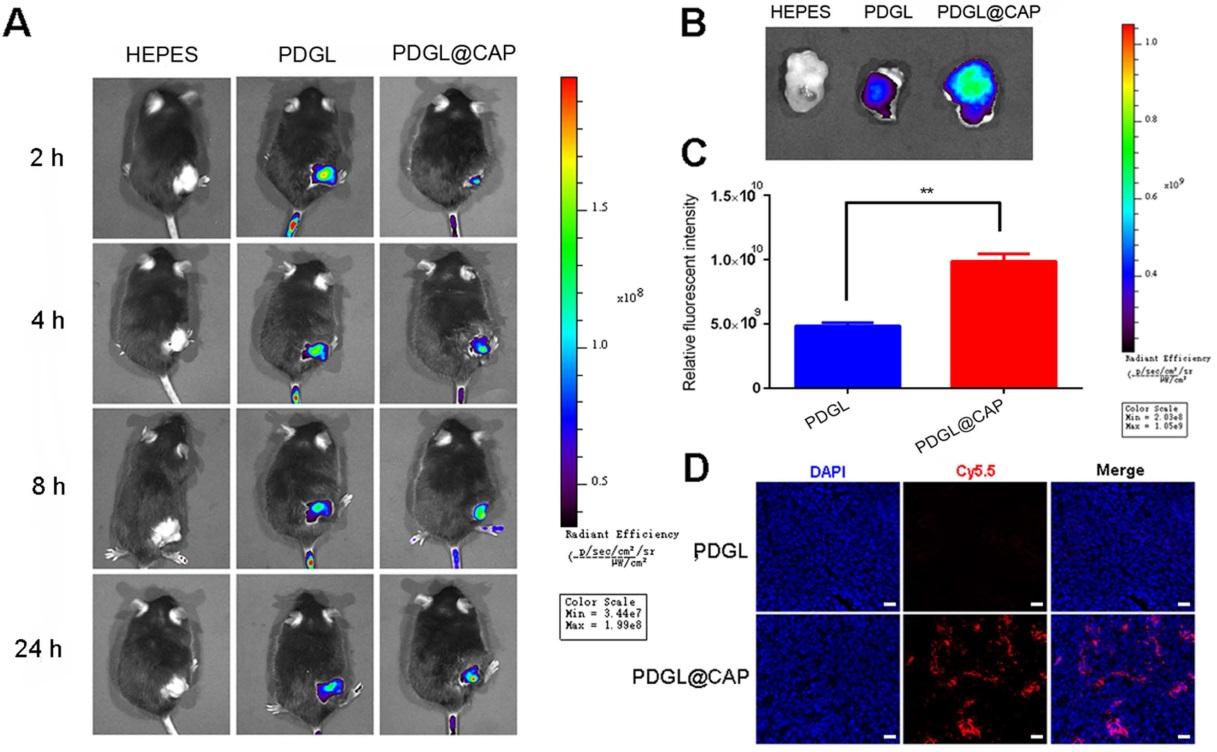
Biodistribution of Cy5.5-labeled nanoparticles in mice bearing Pan 02 xenografts. Nanoparticles were injected systemically. **(A)** *In vivo* images at different time points. **(B)** *Ex vivo* images of tumor tissues dissected at 24 h after injection. **(C)** Quantitation of the results in panel (B) (n = 3, mean ± SD). ** *p* < 0.01. **(D)** Confocal micrographs of tumor sections. Scale bar: 100 µm.

Mice were intravenously injected with Cy5.5-labeled PDGL or PDGL@CAP, then analyzed using an *in vivo* fluorescence imaging system at 1-24 h afterwards. Although PDGL quickly reached the tumor, its accumulation there peaked at 2 h, then declined gradually (Figure 5A-C). In contrast, PDGL@CAP accumulation peaked at 8 h, and levels in the tumor were 2.44 times higher than those of PDGL at 24 h. The greater tumor accumulation of PDGL@CAP was confirmed in tumor sections (Figure 5D). These results may mean that the larger PDGL@CAP can engage in stronger enhanced permeability and retention effects with the tumor. At the same time, CAP encapsulation reduced nanoparticle levels in kidney at 24 h (Figure S8). These results support the idea that our nanoparticles serve as TME-responsive, size-shrinkable nanobombs that initially are large enough to avoid rapid clearance and to accumulate in the TME, where they then become small enough to penetrate deeply into tumors.

### Anti-tumor efficacy of PDGL-GEM@CAP/CQ against PDAC xenografts *in vivo*

**Figure 6:**
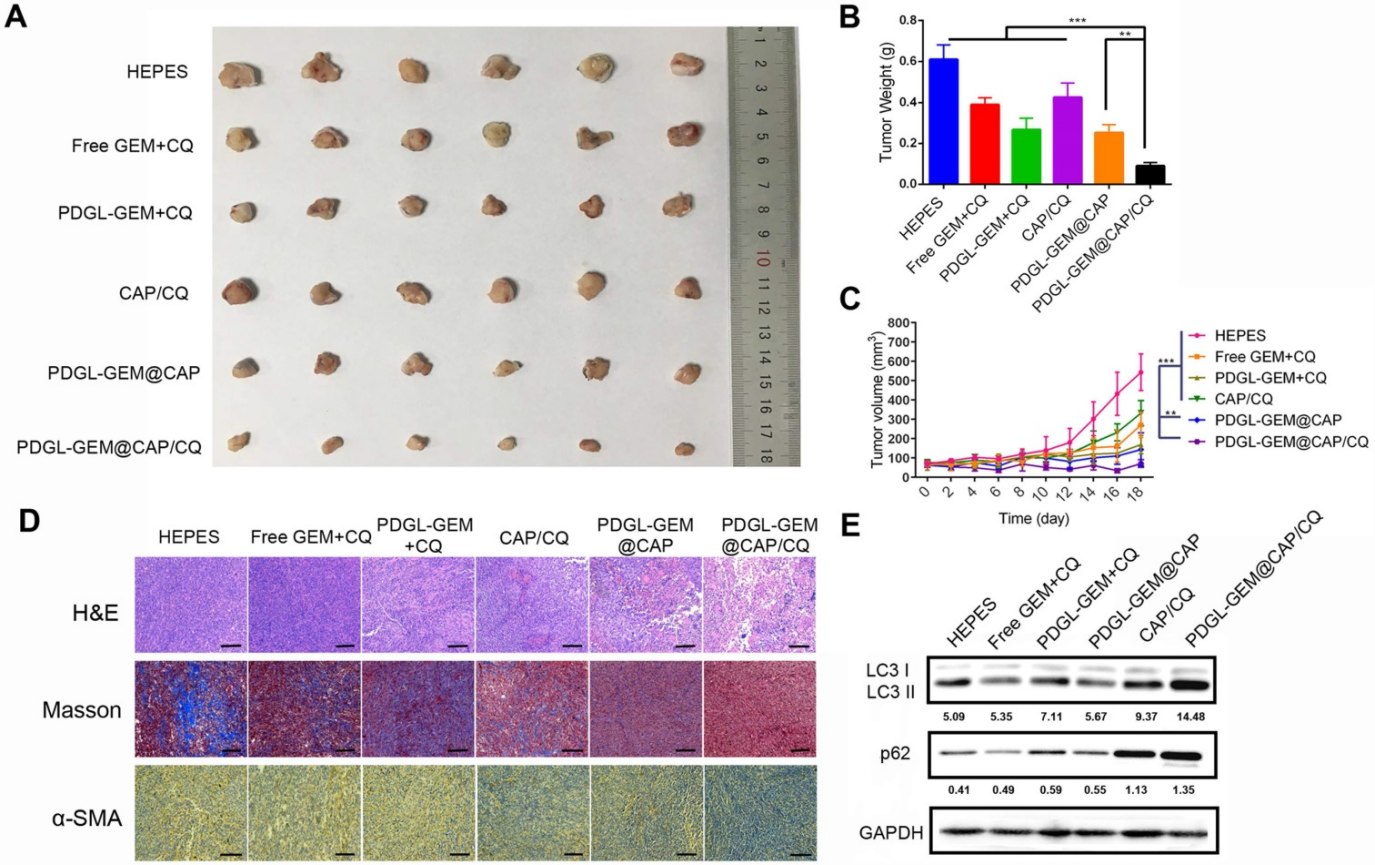
Ability of different nanoparticles to inhibit Pan 02 xenograft growth in mice. **(A)** Photographs and **(B)** weights of tumors at day 18 after the first treatment. **(C)** Tumor volume (mean ± SD, n = 7). **(D)** Tumor slices stained with hematoxylin-eosin (H&E), Masson blue or antibody against α-SMA. Scale bar: 100 µm. **(E)** Levels of LC3 I, LC3 II and p62 in tumor tissue, as detected by western blot. Relative expression levels beneath the gel images were determined using Image J. Levels of p62 were normalized to those of GAPDH, while levels of LC3 II were normalized to those of LC3 I. ** *p* < 0.01, *** *p* < 0.001.

While all nanoparticles showed some anti-tumor activity against Pan 02 xenografts in mice, PDGL-GEM@CAP/CQ exhibited the strongest effects, suggesting that co-delivered CQ and GEM worked synergistically (Figures 6A-C).

PDGL-GEM@CAP/CQ and PDGL-GEM@CAP/CQ caused obvious necrosis in tumors (Figure 6D), and both PDGL-GEM@CAP/CQ and CAP/CQ significantly reduced amounts of ECM collagen. In fact, CAP/CQ, PDGL-GEM@CAP and PDGL-GEM@CAP/CQ reduced the number of activated fibroblasts in the TME, which were identified based on their immunopositivity for α-SMA. PDGL-GEM@CAP/CQ reduced the number of activated cells to the greatest extent. These results suggest that the combination of CQ and GEM reduces fibrosis in tumor tissue by inhibiting activation of tumor-related fibroblasts in the TME. The maximal anti-tumor efficacy of PDGL-GEM@CAP/CQ correlated with the strongest inhibition of autophagy in xenograft tissues, based on the ratio of LC3 II to LC3 I and the level of p62 (Figure 6E).

PDGL-GEM@CAP/CQ appeared to be safe for the animal, based on analysis of body weight during administration (Figures S9) and lack of obvious pathology in slices of liver, heart, lung, kidney, and spleen after staining with hematoxylin-eosin (Figure S10).

### Anti-tumor and anti-metastatic efficacy of PDGL-GEM@CAP/CQ against orthotopic PDAC tumors *in vivo*

**Figure 7:**
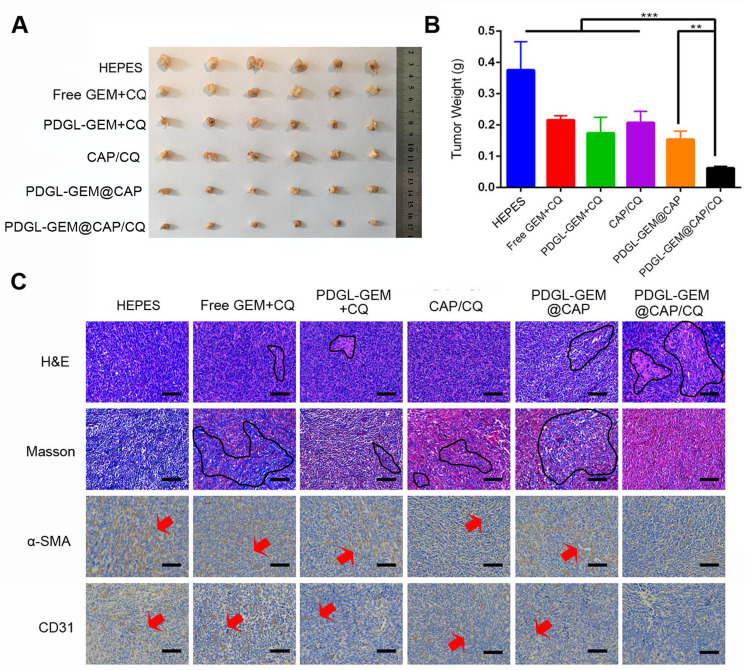
Ability of different nanoparticles to inhibit Pan 02 orthotopic tumor growth in mice. **(A)** Photographs and **(B)** weights of tumors on day 18 after the first treatment (mean ± SD, n = 7). (C) Tumor slices stained with hematoxylin-eosin (H&E), Masson blue or antibody against α-SMA. Scale bar: 100 µm. ** *p* < 0.01, *** *p* < 0.001.

To analyze the effects of PDGL-GEM@CAP/CQ on PDAC metastasis, we grew orthotopic Pan 02 tumors in mice. Such tumors metastasize primarily to the septum and mesentery, which reasonably mimics the clinical situation, in which more than 80% of cases of locally advanced pancreatic cancer metastasize to the peritoneal/omentum and liver 30, 31. Similar to the results in the xenograft model, PDGL-GEM@CAP/CQ showed the strongest anti-tumor effects (Figure 7A-B) and it induced the greatest extent of necrosis as well as the greatest inhibition of fibroblast activation (Figure 7C). In fact, PDGL-GEM@CAP/CQ also inhibited tumor neovascularization based on immunolabeling for CD31.

**Figure 8:**
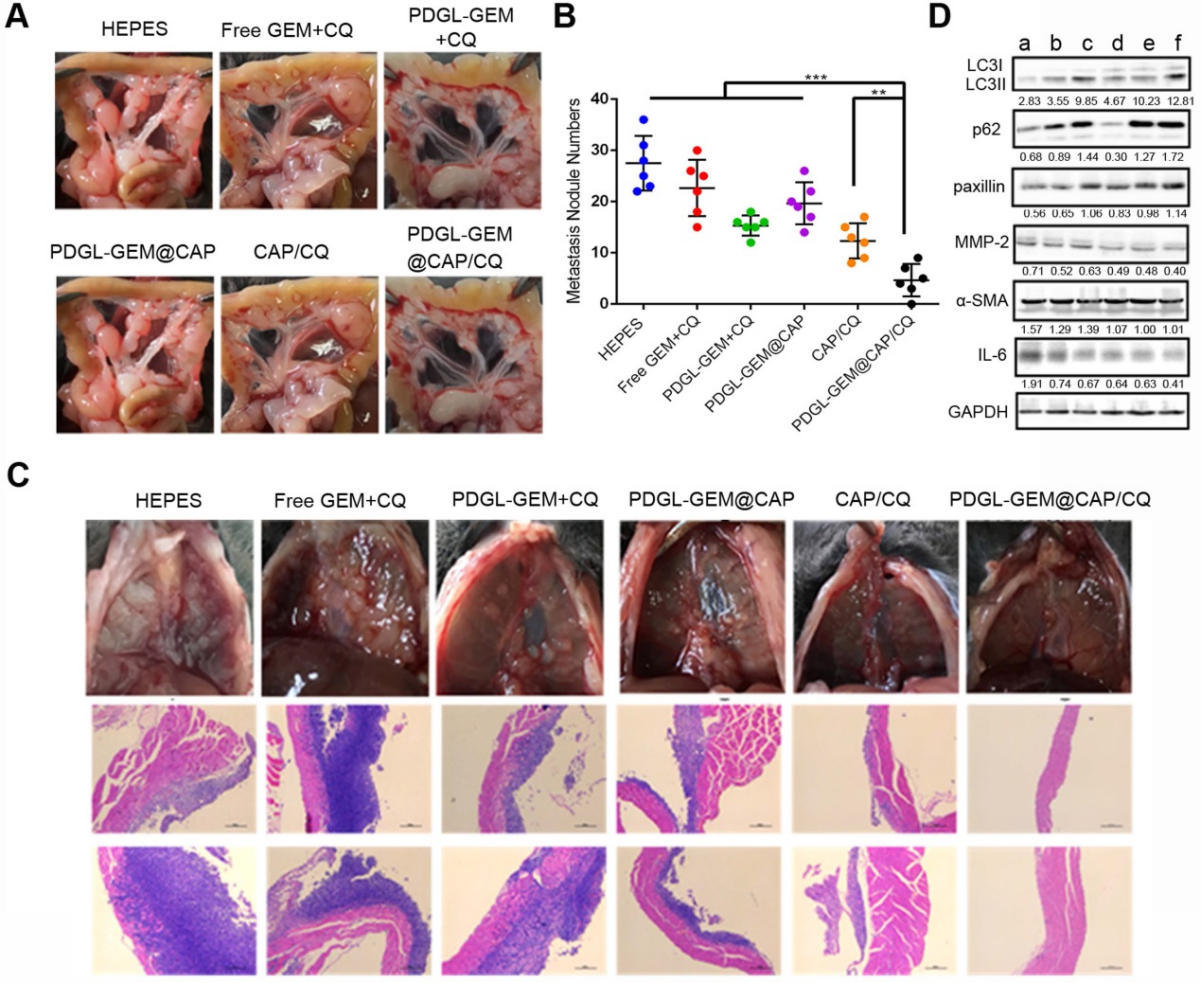
** (A)** Photographs of mesentery of Pan 02 orthotopic tumor-bearing mice on day 18 after the first treatment. **(B)** Quantitative analysis of globular tumor nodules in the mesentery vessels and diaphragm (n = 6). **p < 0.01, ***p < 0.001. **(C)** Representative images and H&E staining of thoracic diaphragm of Pan 02 orthotopic tumor-bearing mice on day 18 after the first treatment. **(D)** Expression of LC3, p62, paxillin, MMP-2, ɑ-SMA, and IL-6 in orthotopic Pan 02 tumors, as detected by western blot. a: HEPES, b: Free GEM + CQ, c: PDGL-GEM + CQ, d: PDGL-GEM@CAP, e: CAP/CQ, f: PDGL-GEM@CAP/CQ. The numbers represent the gray values measured by image J and normalized to GAPDH. LC3 II was further normalized to LC3 I.

Mice treated with HEPES showed a large number of globular tumor nodules along the mesentery vessels and diaphragm (Figure 8A-B). Animals treated with PDGL-GEM@CAP/CQ showed smooth and flat mesentery and diaphragms with very few metastatic nodules, better than the results obtained with CAP/CQ or PDGL-GEM@CAP (Figure 8B-C). Micrometastasis was observed in liver slices from mice treated with any of the formulations except CAP/CQ or PDGL-GEM@CAP/CQ (Figure S11).

Consistent with our *in vitro* experiments, we found that PDGL-GEM@CAP/CQ led to the highest ratio of LC3 II to LC3 I and highest level of p62 in orthotopic tumors (Figure 8D), indicating maximal inhibition of autophagy at tumor sites. PDGL-GEM@CAP/CQ also preserved paxillin levels while down-regulating MMP-2 and IL-6 as well as inhibiting activation of tumor-associated fibroblasts (Figure 8D). These results provide further *in vivo* support for the idea that PDGL-GEM@CAP/CQ inhibits fibrosis in pancreatic tumors and reduces communication between tumor cells and adjacent fibroblasts, which is important for tumor growth and metastasis 32.

To support the fast tumor growth, the autophagic level in TME of pancreatic cancer is upregulated. Autophagy is required for tumor metastasis by degrading paxillin and disassembling focal adhesion 33. Moreover, MMPs, which degrade extracellular matrix, also play key roles in cells invasion, especially in tumor cells with high autophagic levels 34. Therefore, autophagy inhibition may remodulate TME and enhance the anti-tumor and anti-metastasis effects of chemotherapy. The formulated pH sensitive size-shrinkable nanobomb PDGL-GEM@CAP/CQ is capable of overcoming the stromal barrier by responding to the acidic TME, disassembling into small PDGL-GEM and penetrating into the deeper region of tumor. Moreover, the released CQ downregulates the autophagic level in both stromal cells and tumor cells. The sequential delivery of CQ and chemotherapeutic GEM offers a solution to overcome the stromal barrier in pancreatic cancer in both physical and molecular level.

To further assess the toxicity of our nanoparticles, we examined aspartate transaminase, alanine aminotransferase and blood urea nitrogen levels in treated mice bearing orthotopic tumors. While all three indicators were elevated in animals treated with PDGL-GEM + CQ, none was elevated in animals treated with the other formulations. This suggests that the high zeta potential of PDGL can cause toxicity, which is nevertheless absent from the final PDGL-GEM@CAP nanoparticles (Figure S12). As in the xenograft model, PDGL-GEM@CAP/CQ did not show signs of systemic toxicity, based on analysis of body weight (Figure S13) or hematoxylin-eosin staining of slices of heart, lung, kidney and spleen (Figure S14). These results suggest that PDGL-GEM@CAP/CQ is a safe drug delivery system.

## Conclusions

We have constructed an acid-sensitive, size-switchable CAP nanoparticle system loaded with the autophagy inhibitor CQ and the chemotherapeutic GEM. The large nanoparticles are efficiently retained in the TME, where the low pH triggers dissolution of the CAP shell and release of the PDGL-GEM and CQ deep into tumors. The two drugs work synergistically to inhibit tumor growth and metastasis, in part by inhibiting autophagy, which in turn suppresses tumor fibrosis and down-regulates MMP-2, further inhibiting metastasis. This system may provide a new paradigm for pancreatic cancer treatment.

## Materials and methods

### Materials

Gemcitabine hydrochloride was purchased from Meilun Biological Technology (Dalian, China). Chloroquine diphosphate (CQ) and phosphate-PEG_5000_-NH_2_ (PEG_5K_) were purchased from Sigma-Aldrich (USA). The third-generation dendrimer poly-lysine (DGL-G3) was purchased from Colcom (France). Succinic anhydride (SA), 6-phosphonohexanoic acid (6PA), 2-propyl-3-methyl maleic anhydride (CDM) and 4-dimethylaminopyridine (DMAP) were purchased from TCI Chemicals (Shanghai, China). N-hydroxy-succinimide (NHS), 1-[3-(dimethylamino) propyl]-3-ethylcarbodiimide hydrochloride (EDC), ultra-dry N-N-dimethylformamide (DMF), ultra-dry dimethyl sulfoxide (DMSO) and hydrazine hydrate were obtained from J&K Scientific (Beijing, China). Cy5.5-NHS was purchased from Lumiprobe (USA). 1,1'-dioctadecyl-3,3,3',3'-tetramethylindodicarbocyanine (DiD) and 3-(4,5-dimethyl-2-tiazolyl)-2,5-diphenyl-2H-tetrazolium bromide (MTT) were obtained from Beyotime Biotechnology (Beijing, China). 4,6-diamidino-2-phenylindole (DAPI) was purchased from Solarbio Science & Technology (Beijing, China). Low-melting-point agarose was purchased from Gibco (USA). Horseradish peroxidase-conjugated goat anti-rabbit lgG as well as rabbit primary antibodies against GAPDH, paxillin, LC3, p62, MMP2, CD31, α-SMA, and IL-6 were obtained from Abways (Shanghai, China). All other chemicals were of analytical grade.

### Cell lines and animals

The mouse pancreatic cancer cell line Pan 02 and mouse embryonic fibroblast cell line NIH3T3 were obtained from the Cell Bank of Typical Culture Preservation Committee (Chinese Academy of Sciences, Shanghai, China). The cells were maintained in high-glucose DMEM (Hyclone, USA) supplemented with 10% fetal bovine serum (FBS, Gibco, USA) in a 5% CO_2_ atmosphere.

C57BL/6J mice were obtained from Dashuo Biotechnology (Chengdu, China). All animal experiments were conducted under the guidance of the Experimental Animals Administrative Committee of Sichuan University.

### Synthesis of DGL-GEM

GEM-DGL was conjugated as previously reported by our group 25. Briefly, GEM·HCl (20 mg) was dissolved in 1 mL ultra-dry DMSO and reacted with triethylamine at 30 °C under N_2_ for ~12 h to obtain GEM. Then, 4-dimethylaminopyridine (DMAP, 5.1 mg) and SA (7.3 mg) were dissolved in DMSO, and the mixture was added to GEM. The reaction was carried out with stirring at 30 °C under N_2_, and was monitored by thin-layer chromatography until carboxyl-modified GEM was obtained.

EDC (20.0 mg) and NHS (11.0 mg) were dissolved in DMSO, then added to the carboxyl-modified GEM and allowed to react at room temperature under N_2_ for 1 h. Afterwards, DGL dissolved in DMSO was added dropwise and reacted with stirring at 30 °C under N_2_ for 40 h. The product was then purified by dialysis for 48 h against ultrapure water in a membrane with a molecular weight cut-off of 3.5 kDa. The resulting GEM-DGL was lyophilized. Conjugation was confirmed by ^1^H-NMR.

### Synthesis of 6PA-DGL-GEM (PDGL-GEM)

6PA was conjugated to the residual amine group of DGL via an amide reaction. 6PA (3.6 mg) was dissolved in DMF, then EDC (3.2 mg), NHS (2.7 mg) and triethylamine (9 μL) were added, and the reaction was allowed to proceed at 35 °C for 2 h under N_2_. The resulting DGL-GEM was dissolved in DMF and added to 6PA, which were allowed to react at 35 ℃ for 36 h with stirring. The reaction was then dialyzed against ultrapure water for more than 48 h to remove unreacted species, and the PDGL-GEM was lyophilized. Blank PDGL was synthesized using the same procedure, except that DGL was used instead of DGL-GEM. The product was confirmed by ^1^H-NMR.

### Preparation of PDGL-GEM@CAP/CQ

This nanoparticle was formulated according to our previous work 12. PDGL-GEM (20 mg), CQ (12.4 mg) and PEG_5K_ (10 mg) were dissolved in 1 mL HEPES-NaCl buffer (50 mM, pH 7.4). An equal volume of Tris-HCl buffer (1 mM, pH 7.4) containing 300 mM CaCl_2_ was then added dropwise at 40 °C with stirring at 1000 rpm. The mixture was incubated for 2 h, then filtered through a 0.22-μm membrane. PDGL-GEM@CAP/CQ was purified on a Sephadex G-50 column. PDGL-GEM + CQ was synthesized using the same procedure, except that Tris-HCl buffer without CaCl_2_ was used.

### Characterization of PDGL-GEM@CAP/CQ

The size and morphology of PDGL-GEM, PDGL-GEM@CAP and PDGL-GEM@CAP/CQ were determined using a Zetasizer Nano ZS90 (Malvern, UK). Samples were stained with 2% phosphotungstic acid and imaged using transmission electron microscopy on a JEM-2100Plus (JEOL, Akishima, Japan). The drug-loading capacity of CQ and GEM were determined using high-performance liquid chromatography.

### Release of GEM and CQ from nanoparticles *in vitro*

The release of GEM and CQ from PDGL-GEM@CAP/CQ was measured in phosphate-buffered saline (PBS) at different pHs. Equal aliquots of PDGL-GEM@CAP/CQ were placed into dialysis bags with a molecular weight cut-off of 8-12 kDa, which were immersed in 50-mL centrifuge tubes containing 25 mL release medium at pH 5.0, 6.5 or 7.4. Samples were incubated at 37 °C with shaking at 90 rpm. The release medium was sampled (1 mL) at predetermined time points (0.5, 1, 2, 4, 8, 12 and 24 h), and 1 mL of fresh medium was added each time to maintain the same total volume. GEM or CQ in the samples was determined by high-performance liquid chromatography, and cumulative release curves were plotted.

### Nanoparticle stability *in vitro*

PDGL-GEM + CQ, CAP/CQ, PDGL-GEM@CAP and PDGL-GEM@CAP/CQ were mixed with HEPES buffer containing 50% fetal bovine serum and incubated at 37 °C with shaking at 75 rpm. Absorbance at 750 nm was measured at 0, 1, 2, 4, 8, 12 and 24 h using a Varioskan Flash Multimode Reader (Thermo Fisher Scientific, USA).

### Hemolysis

DGL, CAP or PDGL@CAP at 1 mg/mL were incubated with PBS containing 2% (v/v) red blood cells from BALB/c mice at 37 °C under stirring at 75 rpm. Triton X-100 (1%) was used as a positive control. At predetermined time points, samples were taken and centrifuged at 1500 rpm for 5 min, then the absorbance at 540 nm of the supernatant was measured using a Varioskan Flash Multimode Reader. The hemolysis ratio was calculated using the formula:

Hemolysis (%) = (A_sample_-A_PBS_)/(A_Triton_-A_PBS_ ) × 100%

Samples at 12 h were also dispersed in PBS and observed under a microscope (DM 2000, Leica, Germany).

### Uptake of nanoparticles by cells in culture

To determine the cellular internalization of different formulations, Cy5.5-NHS was conjugated to PDGL or PDGL@CAP. Pan 02 cells were seeded into 6-well plates (2 × 10^5^ cells/well) and incubated overnight. PDGL-Cy5.5 or PDGL-Cy5.5@CAP (Cy5.5 at 3 μg/mL) were added in the presence of serum-free medium at pH 6.5 or 7.4. After incubation for 4 h, the cells were washed three times with cold PBS, and nanoparticle internalization was observed using confocal laser scanning microscopy (LSM 800, Zeiss, Germany). To quantify internalization, cells were trypsinized and redispersed in PBS for flow cytometry analysis (BD FACS Celesta, USA).

To explore the uptake mechanism, Pan 02 cells were pre-incubated for 2 h at pH 6.5 or 7.4 with chlorpromazine (10 μg/mL) to inhibit clathrin-mediated cellular uptake, nystatin (50 μg/mL) to inhibit caveola-mediated uptake, or amiloride (13.3 μg/mL) to inhibit macropinocytosis. Then the culture medium was removed, and cells were incubated with Cy5.5-labelled PDGL@CAP for another 2 h. Cells were washed three times with ice-cold PBS, trypsinized and analyzed by flow cytometry (BD FACS Celesta, USA).

### Penetration of nanoparticles into tumor spheroids *in vitro*

3D tumor spheroids were created as follows. Briefly, a 96-well plate was first covered with 2% low-melting-temperature agarose. Pan 02 cells were seeded into the agarose inside each well (5.0 × 10^3^ cells/well), and incubated until the diameter of tumor spheroids reached 300 μm. Then, PDGL-Cy5.5 or PDGL-Cy5.5@CAP (Cy5.5 3 μg/mL) was added in medium at pH 6.5 or 7.4, and the plate was incubated for another 8 h. Spheroids were carefully collected, washed four times with ice-cold PBS, fixed with 4% paraformaldehyde, and observed by confocal laser scanning microscopy (LSM 800, Zeiss, Germany) using Z-axis scanning in 10-μm intervals.

### Cytotoxicity

Pan 02 cells were seeded into 96-well plates (1 × 10^4^ cells/well) and incubated overnight. Free GEM + CQ, PDGL-GEM + CQ, PDGL-GEM@CAP and PDGL-GEM@CAP/CQ were incubated for 24 h with cells at pH 6.5 or 7.4. MTT (20 μL, 5 mg/mL) was added to each well and incubated for another 4 h. The medium was replaced with 100 μL DMSO, then plates were incubated at 37 °C with gentle shaking. Absorbance at 490 nm was measured using a microplate reader (Thermo Fisher Scientific, USA). Cell viability was calculated using the formula:

Cell viability (%) = (A_sample_-A_blank_)/(A_control_-A_blank_ ) × 100%

where medium without cells served as the blank, and HEPES-treated cells served as the control.

In addition, the viability of NIH3T3 cells was determined after incubation with PDGL, CAP or PDGL@CAP.

### Autophagic flow

Autophagic flow was determined using mRFP-GFP-LC3 plasmids (Addgene). Pan 02 cells were transfected with plasmid expressing eGFP-LC3 using Lipofectamine 2000 (Thermo Fisher, USA). Pan 02-eGFP-LC3 cells were seeded in 6-well plates (2 ×10^5^ cells/well) and incubated overnight. The cells were treated with CAP/CQ, PDGL-GEM + CQ, PDGL-GEM@CAP/CQ or HEPES at 6.5 or 7.4 for 4 h, fixed with 4% paraformaldehyde, then observed for the presence of eGFP-LC3 puncta (autophagosomes) using confocal laser scanning microscopy (LSM 800, Zeiss, Germany). Autophagosomes were also observed by transmission electron microscopy (JEM-1400 FLASH, JEOL, Japan) in ultrathin sections of Pan 02 cells treated with PDGL-GEM@CAP/CQ at pH 6.5 or 7.4.

In other experiments, Pan 02 cells were treated with a simple mixture of GEM + CQ, PDGL-GEM + CQ, PDGL-GEM@CAP, CAP@CQ or PDGL-GEM@CAP/CQ at pH 6.5, then collected and lysed in the presence of the protease inhibitor PMSF. Western blotting was used to assess the ratio of LC3 Ⅰ to LC3 Ⅱ and the ratio of p62 to GAPDH. Semi-quantitation was performed using Image J (National Institutes of Health, USA).

### Anti-metastatic activity *in vitro*

Pan 02 cells were seeded into 12-well plates (5 × 10^5^ cells/well) and cultured for 24 h. A scratch wound was made using a 200-μL sterile pipette tip. Cells were washed with PBS, then incubated for 48 h with a simple mixture of GEM + CQ, PDGL-GEM + CQ, CAP@CQ, PDGL-GEM@CAP or PDGL-GEM@CAP/CQ (GEM concentration, 0.5 μg/mL; CQ concentration, 2.5 μg/mL) in DMEM (pH 6.5) containing 2% fetal bovine serum. Wound images were captured by microscopy (DM 2000, Lecia, Germany), and wound healing ratios were calculated using Image J.

In a 24-well Transwell system with a pore size of 8 μm, Pan 02 cells were seeded into the upper chamber pre-coated with 50 μL of Matrigel, then treated with the abovementioned formulations for 12 h. Cells that had migrated across the membrane were fixed with 4% paraformaldehyde, stained with 0.2% crystal violet, and observed under an inverted microscope (Leica, Germany). Crystal violet was dissolved in 33% acetic acid, and absorbance at 570 nm was measured using a microplate reader (Spark, Tecan, Switzerland).

### Nanoparticle biodistribution

Mice bearing Pan 02 xenografts were randomized into three groups. When tumor volume reached 100 mm^3^, animals were intravenously injected with Cy5.5-labeled PDGL, PDGL@CAP, or HEPES buffer. At predetermined timepoints, live images were captured using the IVIS^®^ Lumina III (PerkinElmer, MA, USA). Mice were then sacrificed by cervical dislocation, and major organs and the tumor were harvested and imaged *ex vivo*. Tumors were also sectioned, stained with DAPI and observed by confocal laser scanning microscopy (LSM 800, Zeiss, Germany).

### Anti-tumor activity *in vivo*

Mice bearing Pan 02 xenografts were randomized into six groups (7 animals each). When tumor volume reached ~70 mm^3^, animals were intravenously injected every other day, for a total of four times, with a simple mixture of GEM + CQ, PDGL-GEM + CQ, CAP/CQ, PDGL-GEM@CAP, PDGL-GEM@CAP/CQ or HEPES (pH 7.4). The dosage of GEM was 3 mg/kg and the dosage of CQ was 15 mg/kg. Tumor volumes and body weights were monitored. On day 18 after first administration, mice were sacrificed, then tumors and major organs were collected. Tissue sections were stained with hematoxylin and eosin (H&E), antibody against α-SMA, or Masson stain to determine the level of collagen-1. In addition, tumor tissue was lysed and levels of LC 3 and p62 were evaluated by western blot, with GAPDH serving as a reference.

To complement this xenograft model, we established an orthotopic model in mice by injecting Pan 02 cells into the tail of the pancreas (1 × 10^6^ cells per animal). Seven days later, mice were divided into six groups (n = 7) and given different formulations as described above. Body weights were monitored. On day 18 after injection, mice were sacrificed, and tumors as well as major organs were harvested, sectioned, and stained using H&E, Masson stain or antibody against α-SMA or CD31.

Nodules in the mesentery of treated animals were counted, and liver sections were stained with H&E and analyzed for signs of micrometastasis. Tumor tissues were lysed and levels of LC 3, p62, paxillin, MMP-2, α-SMA and IL-6 were evaluated by western blot.

### Biosafety

Mice were treated with PDGL-GEM + CQ, CAP/CQ, PDGL-GEM@CAP, PDGL-GEM@CAP/CQ or HEPES for five consecutive days (n = 5). The dosage of GEM was 3 mg/kg and the dosage of CQ was 15 mg/kg. On day 6 after the last treatment, mice were sacrificed, and levels of aspartate transaminase, alanine aminotransferase and blood urea nitrogen were assayed using a commercial analyzer (Jiancheng, Nanjing, China) according to the instructions of the manufacturer.

### Statistical analysis

All data were presented as mean ± standard deviation (SD). Differences between two groups were assessed for significance using the two-tailed Student's t-tests; differences among more than two groups were assessed using one-way ANOVA with the Tukey post hoc test (Graphpad Prism software). Differences were considered significant if they were associated with p < 0.05 (marked * in figures), p < 0.01 (**) or p < 0.001 (***).

## Supplementary Material

Supplementary figures.

## Figures and Tables

**Table 1:** The size distribution and zeta potential of PDGL-GEM, PDGL-GEM@CAP and PDGL-GEM@CAP/CQ

	Size (nm)	PDI	Zeta potential (mV)
PDGL-GEM	33.5 ± 1.15	0.287 ± 0.024	+ 10.8 ± 1.21
PDGL-GEM@CAP	110.32 ± 3.26	0.191 ± 0.045	+ 5.7 ± 0.25
PDGL-GEM@CAP/CQ	125.25 ± 1.06	0.238 ± 0.016	+ 3.8 ± 0.85

## Abbreviations

CAFs: cancer associated fibroblasts
CAP: calcium phosphate
CLSM: confocal laser scanning microscopy
CQ: Chloroquine phosphate
DAPI: 4,6-diamidino-2-phenylindole
DGL: dendrigraft poly-L-lysine
DiD: 1,1'-dioctadecyl-3,3,3',3'-tetramethylindodicarbocyanine
DLS: dynamic light scattering
DMAP: 4-dimethylaminopyridine
DMF: N-N-dimethylformamide
DMSO: ultra-dry dimethyl sulfoxide
ECM: extracellular matrix
EDC: 1-[3-(dimethylamino) propyl]c-3-ethylcarbodiimide hydrochloride
FBS: fetal bovine serum
GEM: gemcitabine
GFP: green fluorescent protein
HEPES: 4-(2-Hydroxyethyl)-1-piperazine ethane sulfonic acid
H&E: hematoxylin and eosin
HPLC: high performance liquid chromatography
IL-6: interlukin-6
MMP: matrix metalloproteinase
MTT: 3-(4,5-dimethyl-2-tiazolyl)-2,5-diphenyl-2H-tetrazolium bromide
NHS: N-Hydroxy-succinimide
^1^H-NMR: proton nuclear magnetic resonance
6PA: 6-phosphonohexanoic acid
PDAC: pancreatic ductal adenocarcinoma
PDI: polydispersity index
PEG-PCL: poly(ethylene glycol)-poly(caprolactone)
PMSF: phenylmethylsulfonyl fluoride
PSCs: pancreatic stellate cells
RFP: red fluorescent protein
SA: succinic anhydride
α-SMA: α-smooth muscle actin
TEM: transmission electron microscope
TME: tumor microenvironment
TLC: thin layer chromatography
