# Supplementary material for: Co-delivery of autophagy inhibitor and gemcitabine using a pH-activatable core-shell nanobomb inhibits pancreatic cancer progression and metastasis
Source: Theranostics. 2021 Aug 4;11(18):8692–705. doi: 10.7150/thno.60437 (PMC8419034; doi:10.7150/thno.60437)
Supplement: Supplementary file 1 — Supplementary figures. [file thnov11p8692s1.pdf]

Co-delivery of autophagy inhibitor and gemcitabine using a pH-activatable core-shell nanobomb inhibits pancreatic cancer progression and metastasis

Xiaoxiao Chen[#], Yuan Tao[#], Manmin He, Miao Deng, Rong Guo, Qinglin Sheng, Xuhui Wang, Kebai Ren, Ting Li, Xuan He, Shuya Zang, Zhirong Zhang, Man Li*, Qin He*.

Key Laboratory of Drug-Targeting and Drug Delivery System of the Education Ministry and Sichuan Province, Sichuan Engineering Laboratory for Plant-Sourced Drug and Sichuan Research Center for Drug Precision Industrial Technology, West China School of Pharmacy, Sichuan University, Chengdu 610041, People's Republic of China

* Corresponding Author: Qin He, Man Li

# Contributed equally to this work

Email: qinhe@scu.edu.cn (Q. He), manli@scu.edu.cn (M. Li)

Tel/Fax: +86-28-85502532

Key Laboratory of Drug-Targeting and Drug Delivery System of the Education Ministry and Sichuan Province, West China School of Pharmacy, Sichuan University, No. 17, Block 3, Southern Renmin 18 Road, Chengdu 610041, P. R. China.

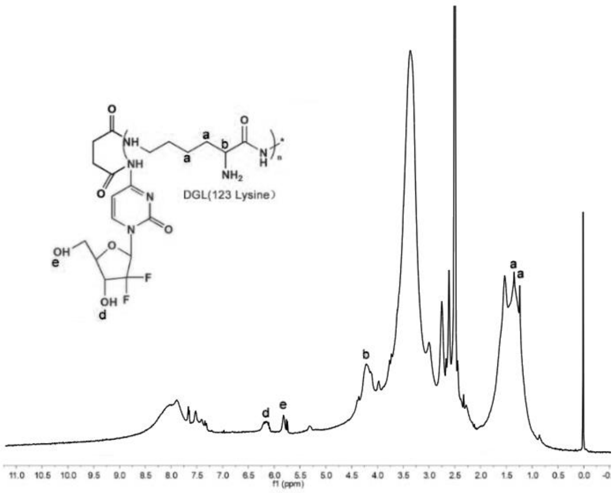

Figure S1. ^{1}H-NMR confirmation of DGL-GEM.

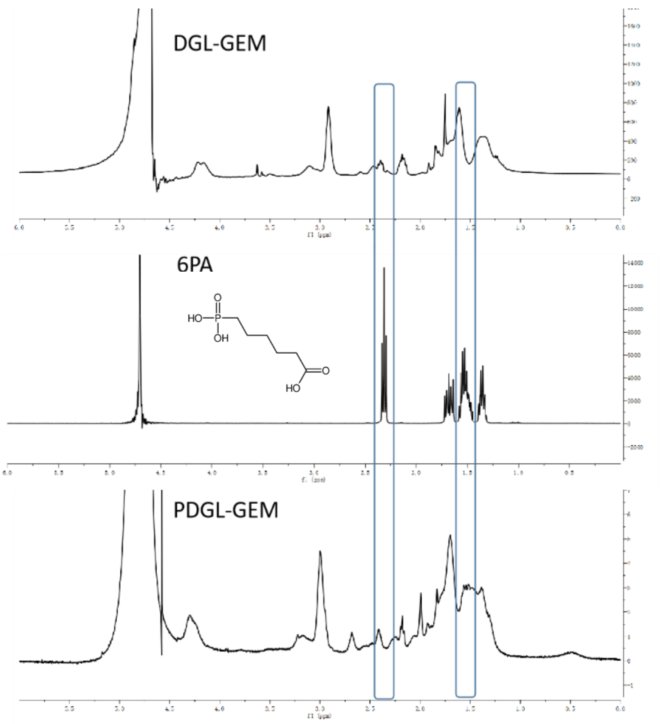

Figure S2. Confirmation of 6PA modified DGL-GEM.

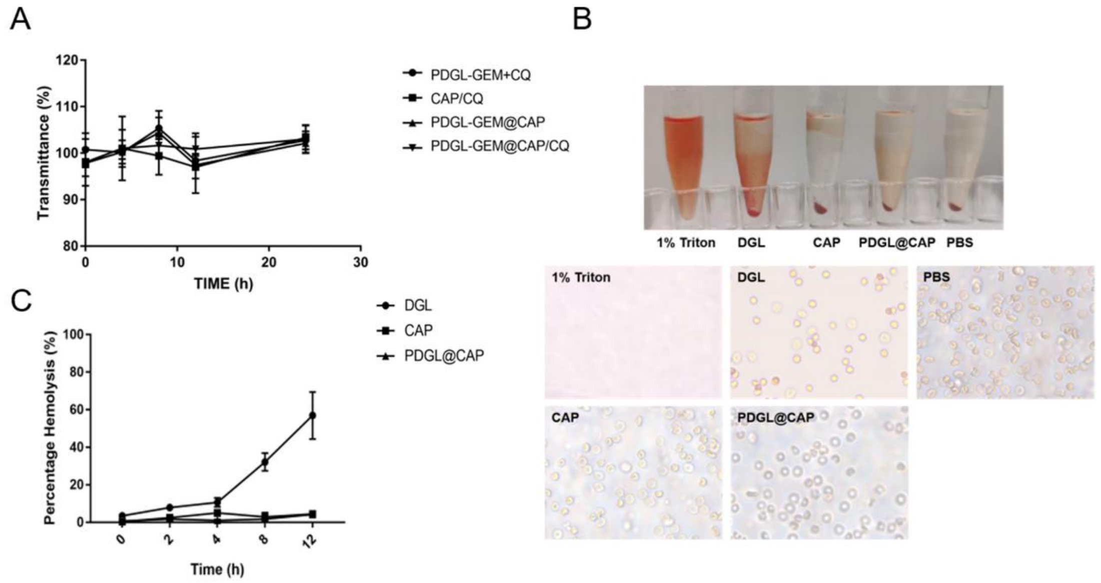

Figure S3 (A) The variations of transmittances of different nanoparticles in 50% FBS (n = 3, mean ± SD). (B) Image of red blood cells incubated with DGL, CAP and PDGL@CAP for 12 h. (C) Time-related hemolysis rates of DGL, CAP and PDGL@CAP for 12 h (n = 3, mean ± SD).

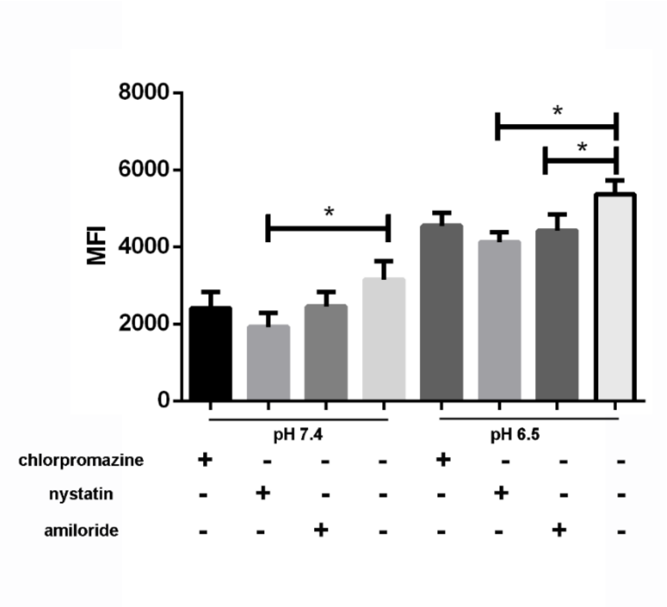

Figure S4. The cellular uptake mechanism of PDGL@CAP (mean ± SD, n = 3. *p < 0.05).

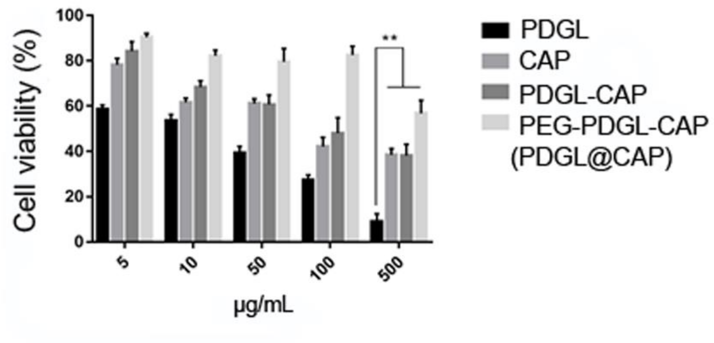

Figure S5. Cell viability of the PDGL, CAP, PDGL@CAP and pegylated PDGL@CAP on NIH3T3 cells (mean ± SD, n=5, **p < 0.01)

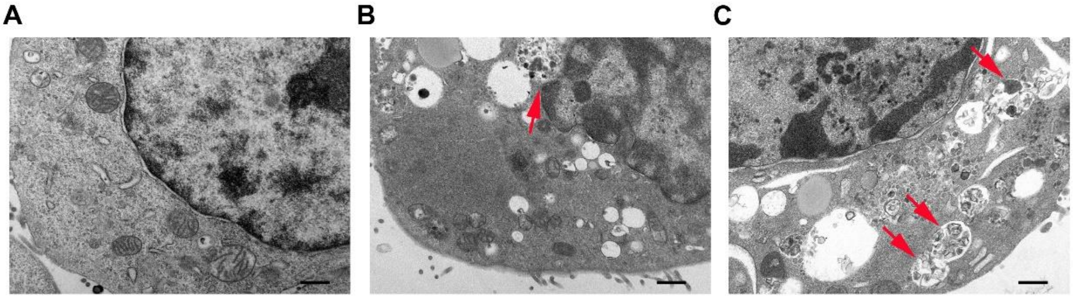

Figure S6. The TEM of Pan 02 cells after treatment with (A) HEPES, (B) PDGL-GEM@CAP/CQ at pH 7.4 and (C) PDGL-GEM@CAP/CQ at pH 6.5. Scale bar = 500 nm.

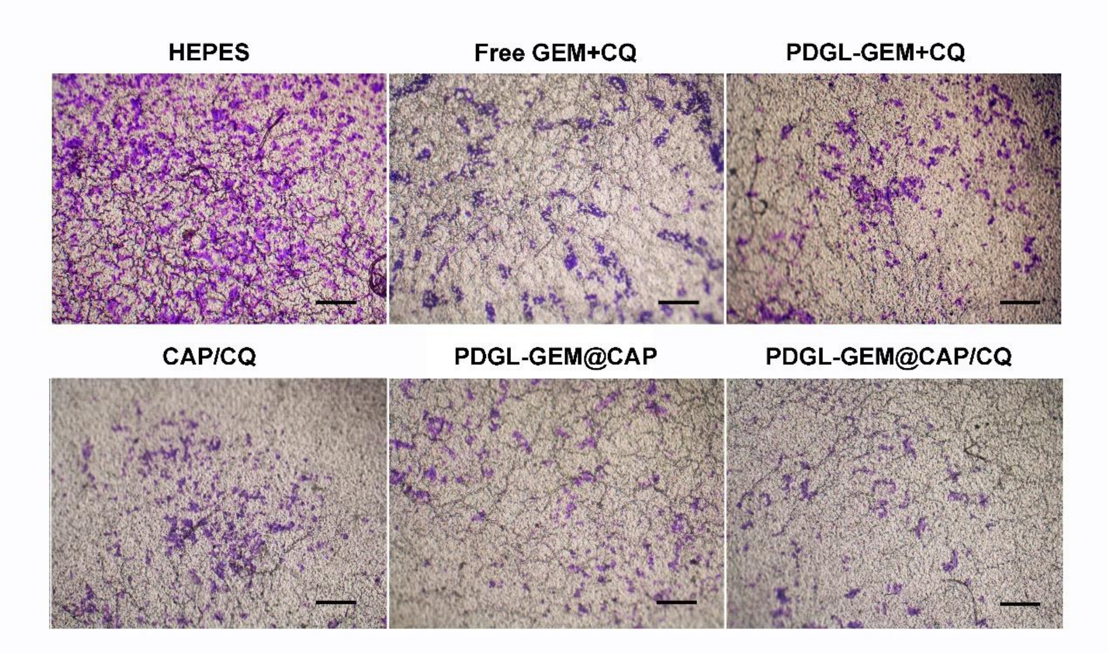

Figure S7. The image of invasion of Pan 02 cells after treatment with different formulation. Scale bar = 100 μm.

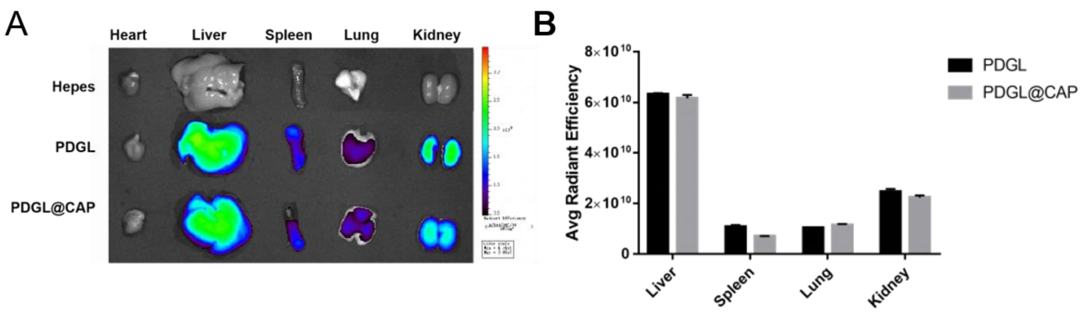

Figure S8. (A) The fluorescence image of ex vivo organs. (B) The semi-quantification results of ex vivo fluorescence in major organs (n = 3, mean ± SD).

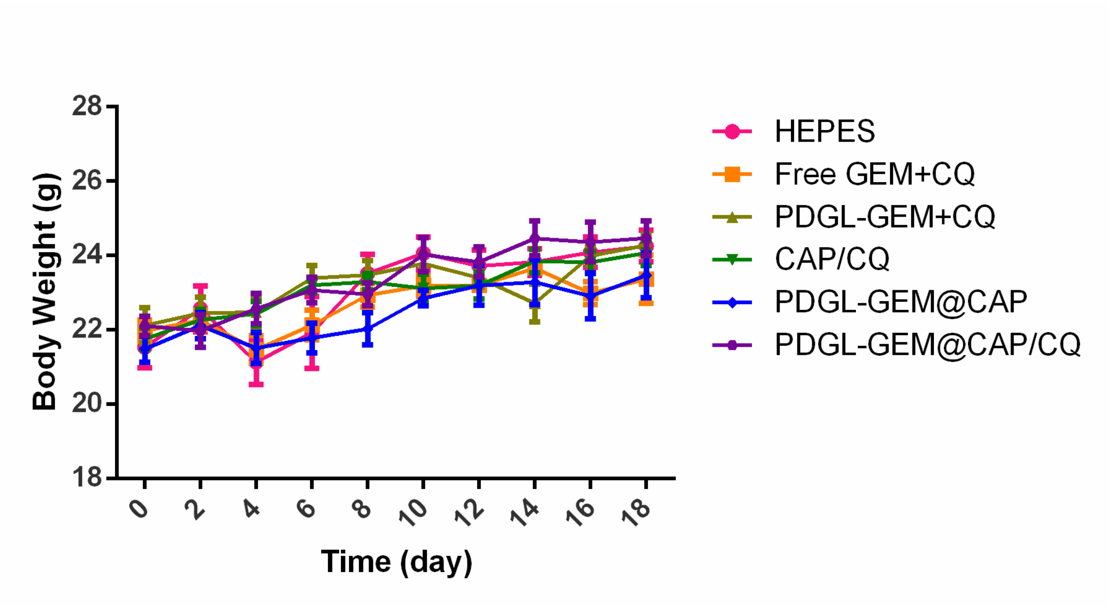

Figure S9. The body weight variation of xenograft Pan 02 tumor-bearing mice (n = 7, mean ± SD).

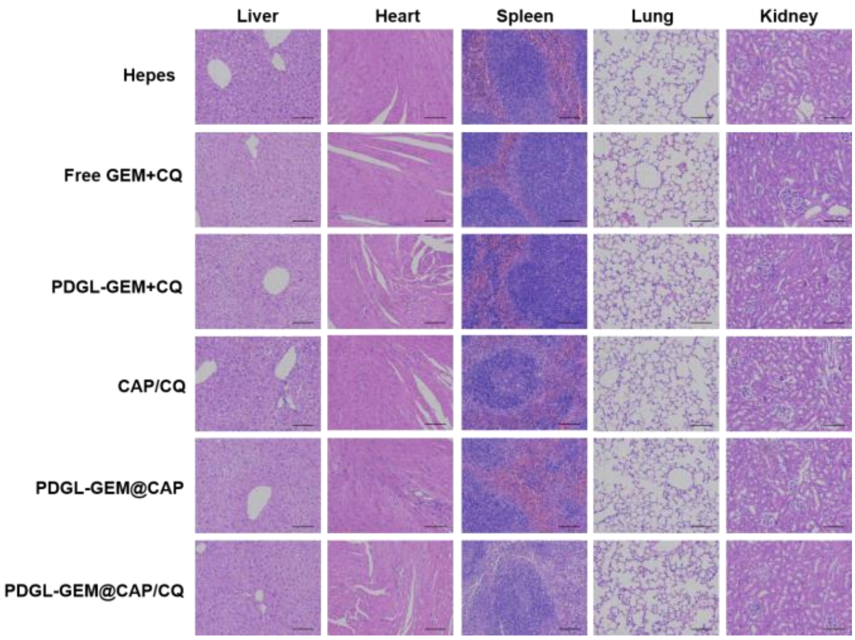

Figure S10. The H&E staining of major organ sections of xenograft Pan 02-bearing mice. Scale bar: 100 μm.

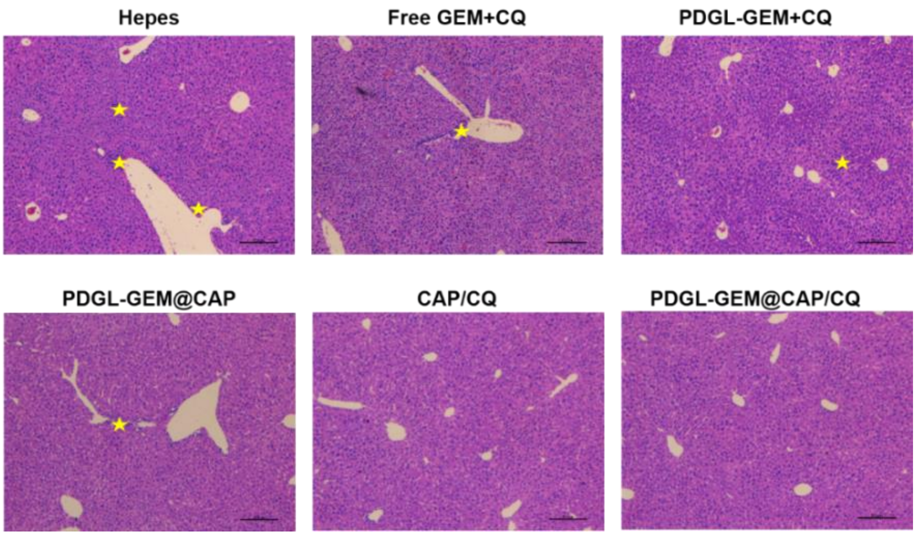

Figure S11. Representative images of liver from each group with H&E staining. Scale bar = 100 μm. Yellow stars indicate the micro metastasis.

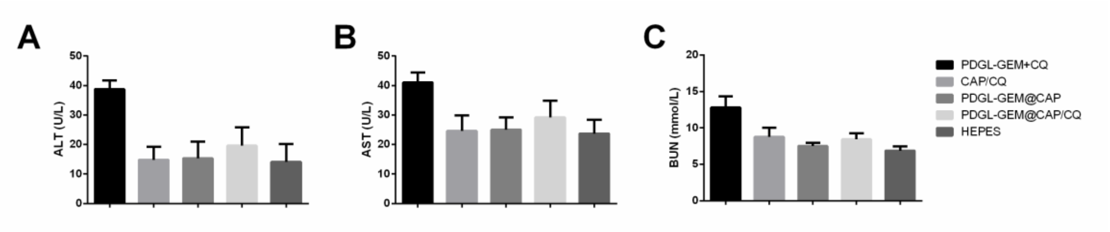

Figure S12. The (A) ALT, (B) AST and (C) BUN level in serum of different formulation treated mice (mean ± SD, n = 3).

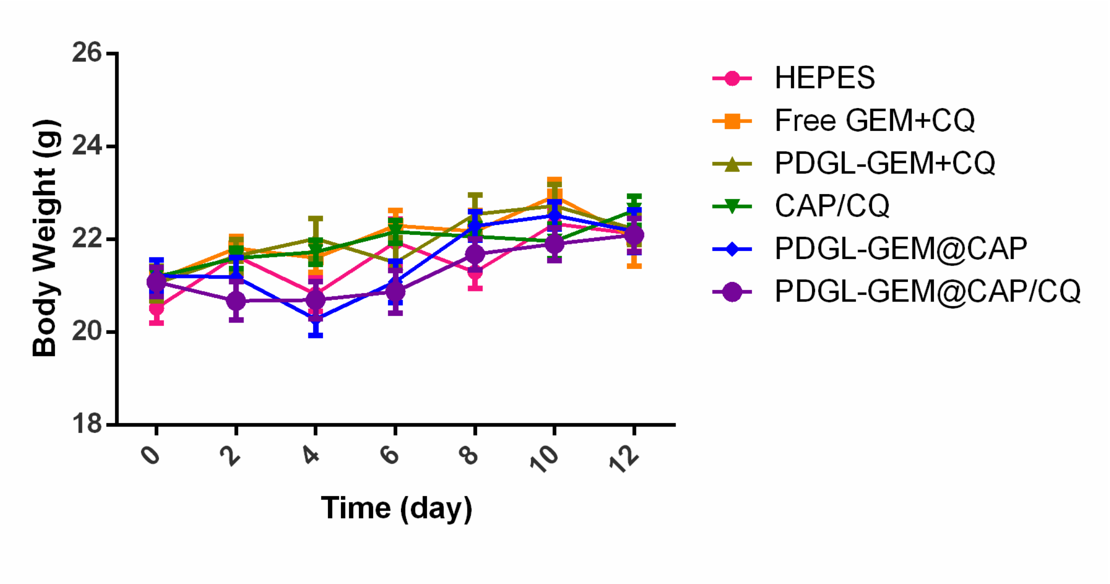

Figure S13. The body weight variation of orthotopic Pan 02 tumor-bearing mice (n = 7, mean ± SD).

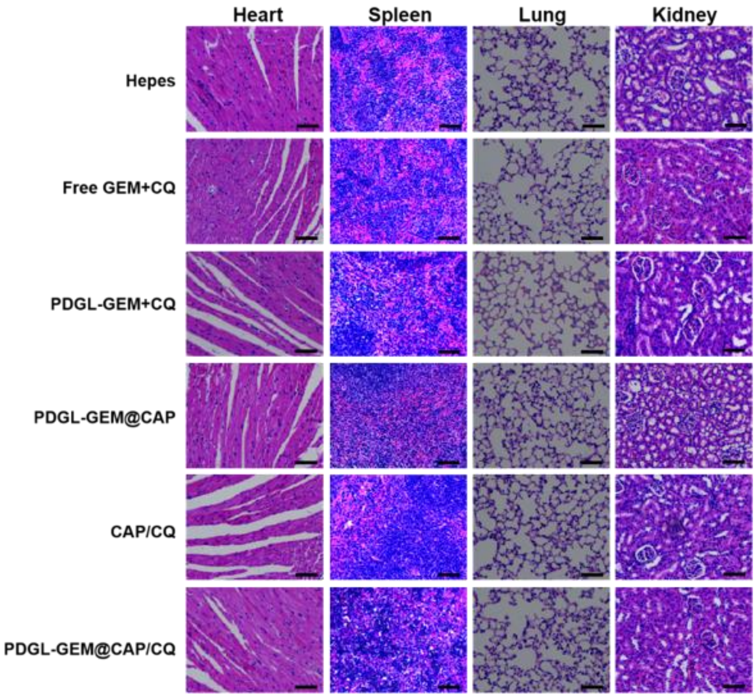

Figure S14. The H&E staining of major organ sections of orthotopic Pan 02-bearing mice. Scale bar = 100 μm.
